# Commentary: Examination of Sensory Recovery of Breasts Reconstructed With Silicone Breast Implants After Nipple-Sparing Mastectomy

**DOI:** 10.1177/22925503231203224

**Published:** 2023-10-04

**Authors:** Rawan ElAbd, Tyler Safran, Joshua Vorstenbosch

**Affiliations:** 1 Plastic Surgery, 54473 McGill University Health Centre, Montreal, Canada

Loss of sensation is common after mastectomy and negatively impacts patient-reported outcomes and satisfaction in alloplastic breast reconstruction following mastectomy.^1,2^ In this prospective cohort study, Sasaki et al examined the sensory recovery of breasts reconstructed with silicone implants after nipple sparing mastectomy by evaluating 9 breast areas. They examined recovery of tactile, pain, and temperature sensation on 31 patients with a follow up of 4 years while comparing different mastectomy incision types.

The authors find that mastectomy significantly decreases all sensation modalities for the implant-reconstructed breast.^3^ Comparing incision types, tactile and pain sensation recovery was significantly better with peri-areolar as compared to inframammary fold (IMF) and lateral incisions in multiple breast regions (*P* < .05). It is worth noting, however, the incision was selected based on tumor localization, and the peri-areolar incision was used for resection of centrally based tumors. This is associated with minimal lateral dissection and trauma to the third through sixth lateral intercostal nerves, which provide sensation to most breast areas, including the nipple areolar complex (NAC), and are frequently overlooked and transected in a mastectomy.^3^ Although the peri-areolar incision might be associated with higher rate of NAC necrosis secondary to circumferential disruption of the blood supply,^4^ this is not necessarily true with sensation, as nerves have the ability to regenerate. Transection of nerves close to the peri-areolar incision site gives them a shorter distance to recover to reinnervate the NAC as compared to IMF or lateral incision.

This article, similar to others, found medial breast sensibility to be better^3^ and discussed that preservation of the internal mammary artery perforator branch could be an indicator of better sensation given the abundance of anterior intercostal nerve perforators accompanying it. This reinforces a very important concept that is usually overlooked by studies assessing breast sensation—the mastectomy flap thickness. Thicker and more perfused flaps theoretically preserve their structural innervation by also preserving the superficial fascial system and efforts of optimizing mastectomy flap thickness when oncologically safe could potentially improve sensation recovery, suggesting that a more anatomic dissection of the breast could yield improved postoperative sensation. This draws parallels to a breast reduction or mastopexy surgical scenario where we regularly observe retention of breast sensation and suggests that sparing these nerves could obviate the need of secondary reinnervation procedures with their associated cost and effect on operating time.^5-7^

Two of the main limiting factors of this study were the absence of a description of mastectomy flap thickness and the pre-operative sensation measurements. As stated above, when discussing sensation of the breast, mastectomy flap thickness represents an important variable to measure. Additionally, without the pre-operative sensation measurements, it is unclear to what extent the sensation truly recovered because the baseline measuements were not reported despite certain variability that exists among patients.

Collectively though we congratulate the authors on conducting this study and sharing their results. It indeed serves as an important guide for future studies on the topic and highlights the prolonged effect of sensory loss on the implant reconstructed breast.
